# Dengue research networks: building evidence for policy and planning in Brazil

**DOI:** 10.1186/s12961-016-0151-y

**Published:** 2016-11-08

**Authors:** Bruna de Paula Fonseca e Fonseca, Fabio Zicker

**Affiliations:** Center for Technological Development in Health (CDTS), Oswaldo Cruz Foundation (Fiocruz), Av Brasil 4036, 8th floor, room 814, Rio de Janeiro, 21040-361 Brazil

**Keywords:** Research networks, Policy and planning, Dengue, Social network analysis

## Abstract

**Background:**

The analysis of scientific networks has been applied in health research to map and measure relationships between researchers and institutions, describing collaboration structures, individual roles, and research outputs, and helping the identification of knowledge gaps and cooperation opportunities. Driven by dengue continued expansion in Brazil, we explore the contribution, dynamics and consolidation of dengue scientific networks that could ultimately inform the prioritisation of research, financial investments and health policy.

**Method:**

Social network analysis (SNA) was used to produce a 20-year (1995–2014) retrospective longitudinal evaluation of dengue research networks within Brazil and with its partners abroad, with special interest in describing institutional collaboration and their research outputs.

**Results:**

The analysis of institutional co-authorship showed a significant expansion of collaboration over the years, increased international involvement, and ensured a shift from public health research toward vector control and basic biomedical research, probably as a reflection of the expansion of transmission, high burden and increasing research funds from the Brazilian government. The analysis identified leading national organisations that maintained the research network connectivity, facilitated knowledge exchange and reduced network vulnerability.

**Conclusions:**

SNA proved to be a valuable tool that, along with other indicators, can strengthen a knowledge platform to inform future policy, planning and funding decisions. The paper provides relevant information to policy and planning for dengue research as it reveals: (1) the effectiveness of the research network in knowledge generation, sharing and diffusion; (2) the near-absence of collaboration with the private sector; and (3) the key central organisations that can support strategic decisions on investments, development and implementation of innovations. In addition, the increase in research activities and collaboration has not yet significantly affected dengue transmission, suggesting a limited translation of research efforts into public health solutions.

**Electronic supplementary material:**

The online version of this article (doi:10.1186/s12961-016-0151-y) contains supplementary material, which is available to authorized users.

## Background

Scientific networking is a critical process for science, technology and innovation (STI) development. Social network analysis (SNA) is a novel method for analysing research output by mapping and measuring relationships between researchers and institutions. The analysis of co-authorship networks through SNA has been used to understand patterns of scientific collaboration [1, 2], evaluate government-funded research programs [3, 4], support policy planning and innovation management in health [5], and global health policy development [6]. As a strategic tool, it provides information for decision-making processes, supporting the performance assessment and development of health science and technology (S&T) organisations [7]. The analysis of co-authorship networks allows identification of opportunities and benefits of collaboration, promoting innovation-related networks in areas where they do not exist [8].

Health research networks are seen as a means to tackle complex problems, which usually require transdisciplinary and multidisciplinary approaches [9]. Networks shape the way problems and solutions are understood, influencing governments, international organisations and other global actors [10]. The contribution of multi-organisational networks to the promotion of health innovations has been discussed, particularly to help developing countries address the challenge of neglected tropical diseases (NTDs) [11, 12]. SNA has been applied to understand scientific collaboration in NTDs and generate evidence to guide policy-planning efforts in Brazil, Canada and Germany [3, 5, 13, 14].

Dengue has been one of the most challenging NTDs due to its rapid geographical expansion. Although new vector control tools have been developed and a dengue vaccine has recently been licensed in a number of countries, the wide impact of these tools in disease transmission is yet to be assessed. A quality-assured point-of-care bedside test for early diagnosis of dengue is also needed to substitute expensive and time-consuming current laboratory tests. Current efforts to curb dengue transmission focus on the vector, but these control efforts have failed to stem the increasing incidence of dengue fever epidemics and expansion of the geographical range of endemic transmission [15, 16]. As a dengue-endemic country, Brazil has sustainably invested in research networks for the development of new tools to control the disease [17]. Although the historical expansion of the disease is well documented in the country, the assessment of the results of these investments, in terms of consolidation, productivity, dynamics and contribution of dengue research networks, has been overlooked.

This paper reports a 20-year retrospective longitudinal evaluation (1995–2014) of the Brazilian national and international dengue research networks, based on the co-authorship of scientific papers, with special interest in institutional collaboration. The study aims to generate evidence on the evolution of scientific connectivity in dengue research that could ultimately inform prioritisation of research, financial investments and health policy.

## Methods

The analysis was based on SNA methods, as described by Fonseca et al. [18]. SNA is a theoretical approach that uses a set of techniques to understand and quantify the relations between members of a network (nodes or actors), which can be individuals, groups, organisations and even whole countries [19]. By quantifying the social structure of a network, namely the set of nodes and their connections, it is possible to identify the most important nodes, the formation of groups, the flow of tangible and intangible resources, among other information [20, 21].

Statistical analysis in SNA includes indicators/metrics that may reflect the properties of the network as a whole or of its individual nodes. The network-level indicators provide information on its overall structure and properties such as size and connectivity. Indicators at the individual level describe the importance of a particular node relative to all other nodes, based on the nature of its interactions. Centrality measures are the most used in SNA to identify the nodes that have strategic significance in the network [18].

In this study, SNA was used to analyse and map co-authorship relations between Brazilian S&T organisations working on dengue research.

### Data collection

The data mining strategy was based on retrieving dengue-related scientific articles, involving Brazilian-based researchers (as authors or co-authors) from 1995 to 2014. The unit of analysis (the nodes in the network) consisted of the organisations where Brazilian-based authors and their national and international collaborators were affiliated at the time of publication.

Three sources of information were used: the Web of Science (WoS), the SciELO (Scientific Electronic Library Online) and the Scopus databases. The SciELO database is originally from Brazil and covers Latin America and Caribbean countries. Queries were directed to the title, abstract and keywords of scientific publications (dengue*) and to the address/affiliation of the authors (brasil OR brazil). Only published articles or articles in press with abstract available were included.

### Data integration and standardisation

Data obtained from the Scopus, SciELO and WoS databases were imported into the data/text mining software VantagePoint (Search Technology Inc.). Database integration and harmonisation into a single dataset was done with the “data fusion” tool of the software.

Scientific papers addressing diseases caused by other flaviviruses or arboviruses, general studies on NTDs that marginally mentioned dengue, and studies on other diseases occurring in dengue endemic areas were excluded from the dataset. The remaining dengue-specific articles were broadly classified according to the subject of research in four major areas: basic biomedical, public health, vector related and clinical research.

Names and acronyms given to a particular organisation were standardised and consolidated, allowing the correct attribution of papers to a specific organisation. The data was processed using the “list cleanup” function of the VantagePoint software on the “Author Affiliations” field.

The organisations participating in the networks were classified into five types, according to their main activities: (1) educational and academic institutions, including those that may or may not carry out research activities; (2) healthcare facilities, which included institutions that provide healthcare services and medical diagnosis; (3) public health institutions, including those directly linked to federal, state or local governments; (4) research institutes, comprising centres engaged in research in several areas of knowledge; and (5) private companies.

### Network assembly, visualisation and analysis

After treatment and processing, the authors’ affiliation data was used to build institutional networks. In these networks, nodes represent organisations, and two or more organisations were connected if their members shared the authorship of one or more papers. Visualisation of the network graphs and statistical analysis of the dataset were produced with the open-source software Gephi [22].

The collaboration dynamics among organisations was assessed through the evolution of co-authorship networks using 5-year interval windows, which allows a more accurate evaluation of their cooperation structure [23, 24]. This approach assumes that the shared authorship of a paper implies a continued collaboration, with a more intensive knowledge exchange within this period [23].

The network structure was described, according to Wasserman & Faust [20], based on the following indicators: number of nodes and links, number of components, size of the giant component, average degree, average path length, and average clustering coefficient. The role of each organisation in the networks was described by four different centrality measures [25]: degree centrality, eigenvector centrality, betweeness centrality, and closeness centrality, each of which quantifies a different aspect of centrality, indicating whether an organisation has a prominent or influential role in the network. In order to obtain a summary Centrality Index (CI), the organisations were first separately ranked according to each of the four centrality measures [26]. The CI corresponded to the sum of their ranking positions. The lowest the CI, the more central an organisation was in the network.

Table 1 provides a theoretical definition of SNA indicators presented herein and their meaning in this study.

**Table 1:** Theoretical definition of social network analysis indicators presented herein and their meaning in this study

Indicator	Definition	Meaning in this study
Network size		
Nodes	Actors within a network	Organisations in the co-authorship network
Links	Relationships or connections between actors	Co-authorship between organisations
Network connectivity/cohesion		
Component	Subset of nodes in a network in which all of them are linked to each other, directly or indirectly	Group of organisations that were connected to one another through joint publications
Giant component	Largest component existing in the network	Largest group of organisations connected through joint publications; the larger the giant component size, or percentage of institutions included within it, the more interconnected the network is
Average degree	Average number of direct connections the network nodes have	Average number of collaborations per organisation; the higher the average degree, the more connected the network is
Average clustering coefficient	Measures the extent to which the nodes in the network establish a perfect cluster, in which all the nodes are interconnected	The extent of full connectivity between organisations; a high average clustering coefficient indicates that more institutions are interconnected within the network
Average path length	Average smallest number of connections that a node needs in order to reach any other in the network	The average distance between organisations; the lower the average path length, the more direct is the connection between organisations
Centrality/significance of nodes in the network		
Degree centrality	Number of a node’s direct connections	A measure of how many direct contacts an organisation has Organisations with high degree centrality are usually focal points of communication in the network
Eigenvector centrality	Reflects the quantity and quality of the direct connections a node has	A measure of high connectivity and links to other highly connected organisations; higher values indicate influential organisations in the network
Betweeness centrality	Indicates to what extent a node acts as a “bridge” between the various other nodes in the network, which would otherwise be disconnected	A measure of how much an organisation mediates the connection between other institutions; an organisation with high betweeness centrality has the potential to control the flow of information in the network
Closeness centrality	Measures how close a node is to all other nodes in the network	A measure of the extent to which an organisation can directly reach others; organisations with high closeness centrality can quickly obtain and communicate information in the network

## Results

**Fig. 1:**
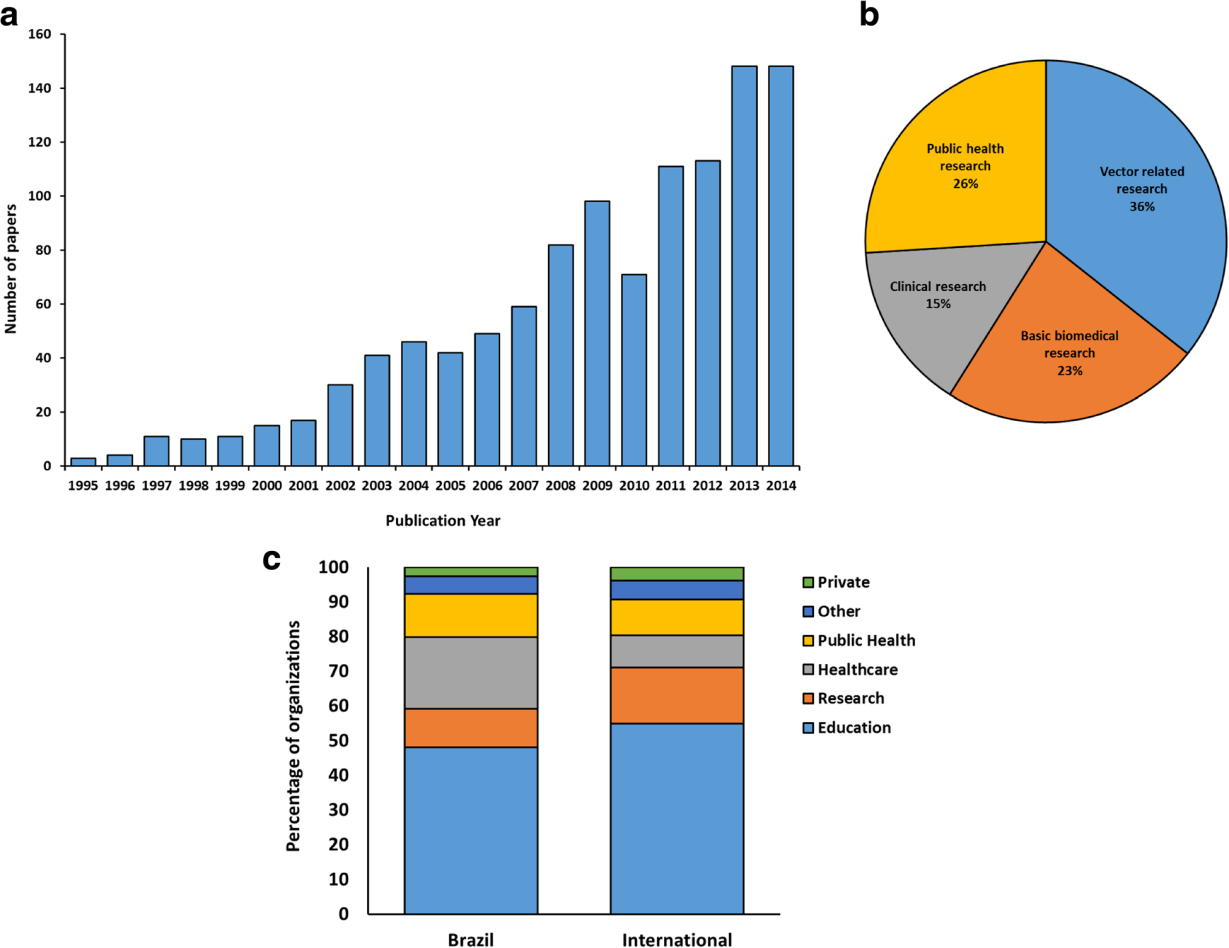
General profile of dengue research involving Brazilian organisations. **a** Annual number of published articles on dengue by Brazilian organisations and their collaborators (1995–2014). **b** Distribution of dengue articles according to the subject area of research. **c** Type of Brazilian and international organisations involved in the dengue research networks

The search retrieved 1076 papers in the WoS, 1153 in Scopus, and 413 in SciELO. After data integration, standardisation and treatment, 1106 unique papers were included in the analysis. The number of dengue articles involving Brazilian organisations has increased over the past 20 years (Fig. 1a); 36 % of the articles were related to dengue vector, including research on vector biology, vector control and virus-vector interactions. Basic biomedical research accounted for 23 % of the publications, including vaccine development, viral biology and basic pathology. Public health research, including epidemiology, represented 26 % of the publications, which were related to epidemic outbreaks, virus circulation, serotyping, morbidity and mortality, epidemic modelling, geographic information systems and cost-analysis studies. Clinical research publications comprised the description of clinical manifestations, pathology, diagnostics and immune responses in general, and corresponded to 15 % of the total publications (Fig. 1b).

The Brazilian network for dengue research involved 298 national and 304 international organisations from 63 countries. Of the Brazilian organisations, 48 % were educational and academic institutions, 12 % were public health institutions, 11 % were research institutes and 21 % were healthcare facilities. A small proportion of private companies (3 %) was also present in the networks. International collaborators had a similar profile, with educational organisations (55 %) also representing a significant part of the networks (Fig. 1c).

### Longitudinal analysis of organisational networks

**Fig. 2:**
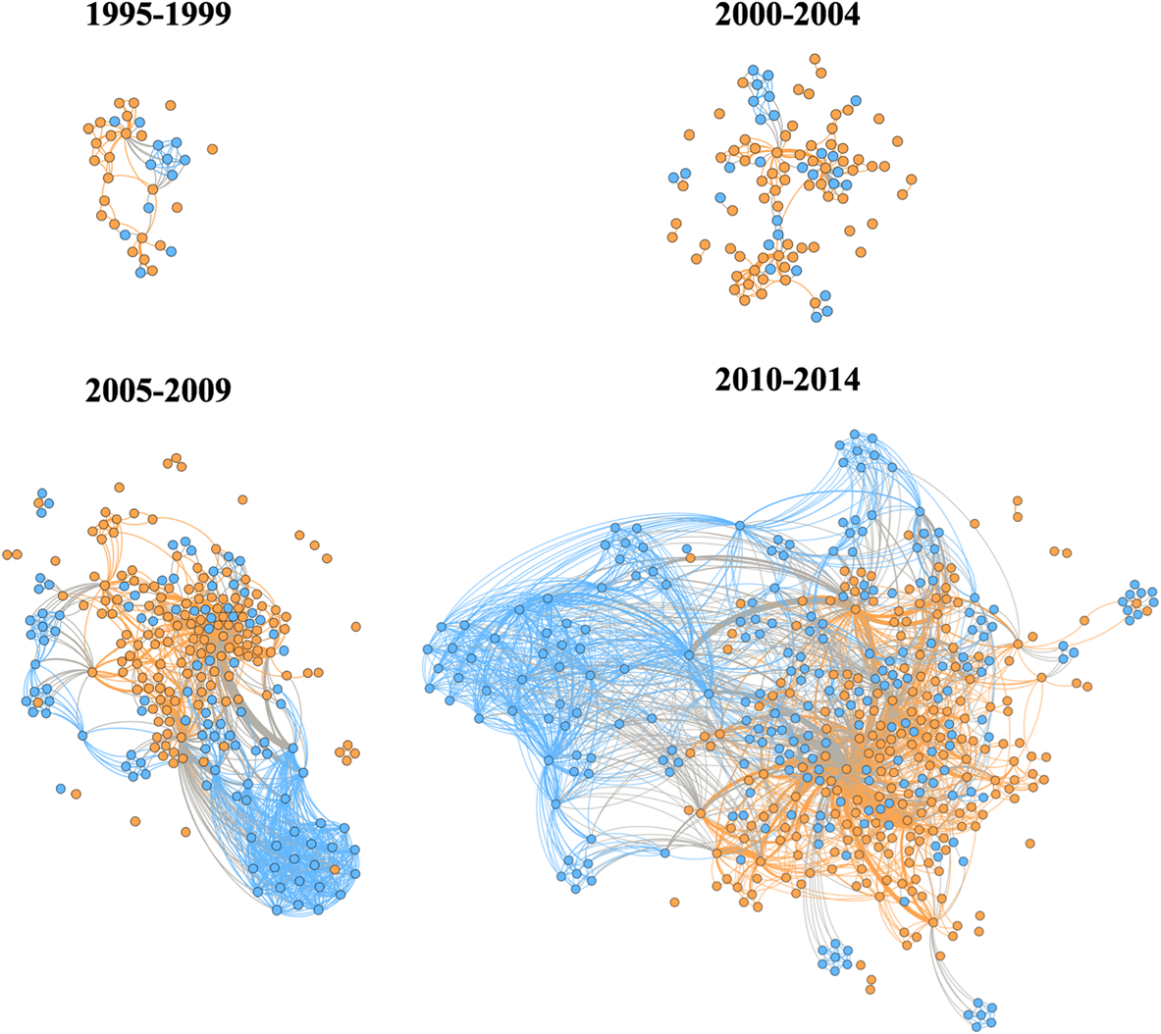
Evolution of the Brazilian collaborative networks on dengue research, 1995–2014. Organisation links were mapped based on the affiliations of the authors of scientific papers. Each node represents one organisation and two organisations were considered connected if their authors shared the authorship of a paper. The thickness of the links indicates the frequency of collaboration between two nodes. The node colour indicates whether the organisation is Brazilian (*orange*) or from abroad (*blue*)

The evolution of dengue research networks in Brazil was analysed in 5-year intervals, as displayed in Fig. 2.

The analysis of the structural characteristics of the networks was based on the indicators shown in Table 2.

**Table 2:** Evolution of the dengue research networks involving Brazilian organisations

Indicator	1995–1999	2000–2004	2005–2009	2010–2014
Number of nodes (organisations)	36	99	254	447
Number of links	72	156	1004	2024
Number of components	4	15	17	12
Giant component size	91.7 %	77.8 %	90.2 %	96.6 %
Average degree	4.3	3.8	8.6	9.35
Average clustering coefficient	0.782	0.753	0.822	0.800
Average path length	2.67	3.44	2.77	2.85

The number of organisations involved in the research networks has considerably increased over the years, especially in the second (2000–2004) and third periods (2005–2009) reviewed. The increase of the giant component size and average degree, associated with the decrease in the number of components, indicated that the network has gained in connectivity through the years. The high average clustering coefficient and low average path length maintained through the entire period corroborate this finding. These results suggest that the network structure was potentially very effective in knowledge generation (high connectedness) and knowledge sharing and diffusion (short distance between members).

**Fig. 3:**
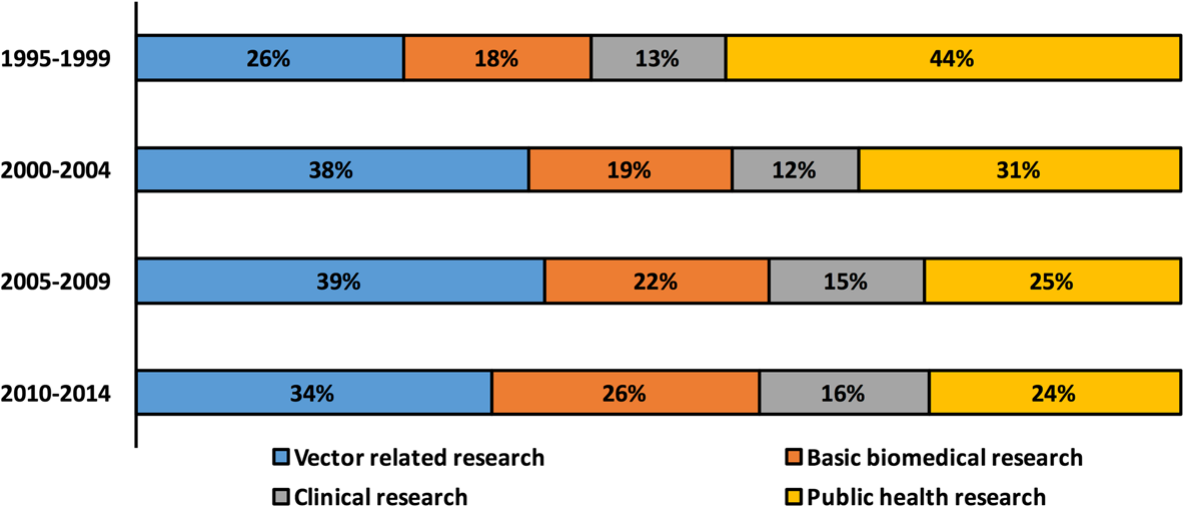
Thematic trends in dengue research involving Brazilian organisations (1995–2014). Dengue-specific articles involving Brazilian organisations were broadly classified according to their subject of research

The network evolution was accompanied by a shift in the research themes. In the first 5 years, public health studies accounted for 44 % of the total research efforts. From the year 2000 onwards, vector-related research gained more importance and became the most frequent theme, followed by a continued increase in basic biomedical research (Fig. 3).

### Centrality analysis

Central organisations usually have greater access and control over resources, leading knowledge exchange and preventing many groups from isolation. Consequently, these institutions are more likely to be associated with innovative activities [27]. The centrality analysis allowed the identification of the most influential Brazilian organisations in the dengue research network in each period (Table 3).

**Table 3:** Most influential Brazilian organisations in the dengue research networks

1995–1999		2000–2004		2005–2009		2010–2014	
Organisations	CI	Organisations	CI	Organisations	CI	Organisations	CI
Fiocruz	4	Fiocruz	5	USP	7	Fiocruz	4
IEC	9	FUNASA	9	Fiocruz	12	USP	15
FUNASA	15	USP	10	UFCE	31	UFMG	16

*CI* centrality index, *Fiocruz* Oswaldo Cruz Foundation, Ministry of Health, *IEC* Evandro Chagas Institute, *USP* University of São Paulo, *FUNASA* National Health Foundation, Ministry of Health, *UFCE* Federal University of Ceará, *UFMG* Federal University of Minas Gerais

The Oswaldo Cruz Foundation and the Federal University of São Paulo were present in most periods, indicating their prominent role in dengue research. The Evandro Chagas Institute, part of the national public health surveillance system, played an important role in the first 5 years evaluated. The National Health Foundation, one of the government institutions responsible for environmental health for disease prevention and control, played a central role in the first and second periods. In the following years, the Federal University of Ceará and the Federal University of Minas Gerais appeared as central organisations in dengue research.

The critical role of these central organisations for the connectivity of the entire network was explored by calculating the network metrics after the exclusion of these institutions (Table 4) and comparing them to the original values (Table 2). The network properties were significantly affected by the removal of central nodes, suggesting that these organisations had a critical role in maintaining network connectivity. More specifically, the number of components greatly increased, the average degree and average clustering coefficient decreased, and the giant component size dropped considerably, especially in the first two periods.

**Table 4:** Dengue research networks excluding key central Brazilian organisations

Indicators	1995–1999	2000–2004	2005–2009	2010–2014
Number of nodes (organisations)	33	96	251	444
Number of links	39	102	811	1663
Number of components	11	35	33	29
Giant component size	36.4 %	17.7 %	80.5 %	90.8 %
Average degree	2.36	3.29	6.46	8.10
Average clustering coefficient	0.680	0.665	0.784	0.759
Average path length	2.33	2.49	3.62	3.49

### International collaboration

**Fig. 4:**
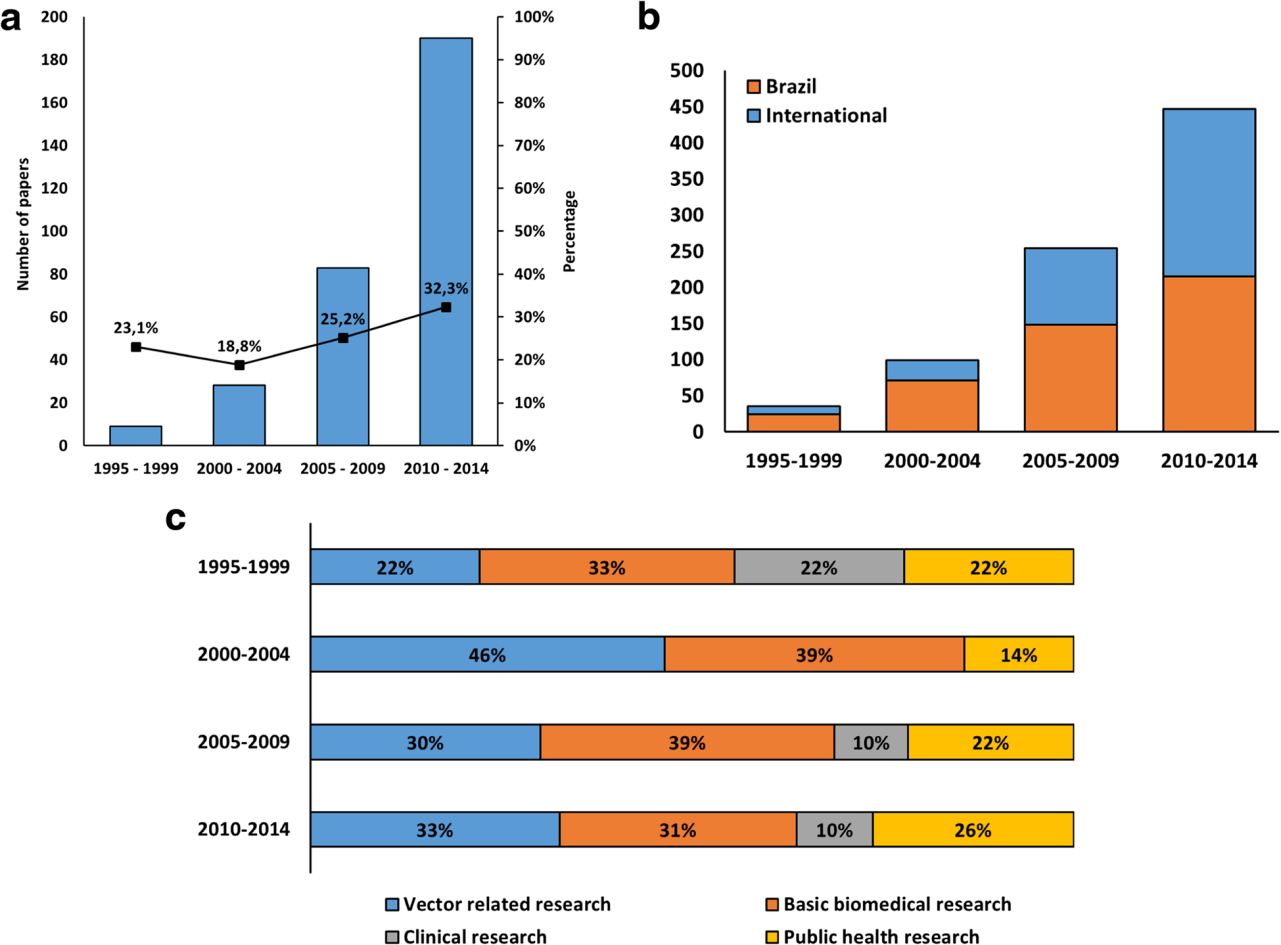
Characteristics of the international collaboration in Brazilian dengue research networks (1995–2014). **a** Number and percentage of papers published in collaboration with international partners. **b** Number of Brazilian and international organisations included in the research networks. **c** Thematic trends in international collaborations

The total number of articles published with international collaboration increased from 23 % to 32 % during the 20 years reviewed (Fig. 4a). Although the overall percentage of international collaborations is still modest, the participation of international institutions in the network has significantly grown throughout the years (Fig. 4b). From the second to the third period there was an approximately four-fold increase in the participation of these organisations in the Brazilian network, which doubled in the last period. However, when foreign institutions were excluded from analysis, the evolution pattern of the Brazilian network structure remained the same (Additional file 1: Table S1), suggesting that the increased connectivity observed over time is an intrinsic pattern of the Brazilian organisations.

Research carried out with international partners interchanged between basic biomedical research, in the first and third periods, and vector-related research, in the second and last periods (Fig. 4c). Consistently, one third of publications with international partners focused on basic biomedical research, but there was limited engagement in clinical research.

**Fig. 5:**
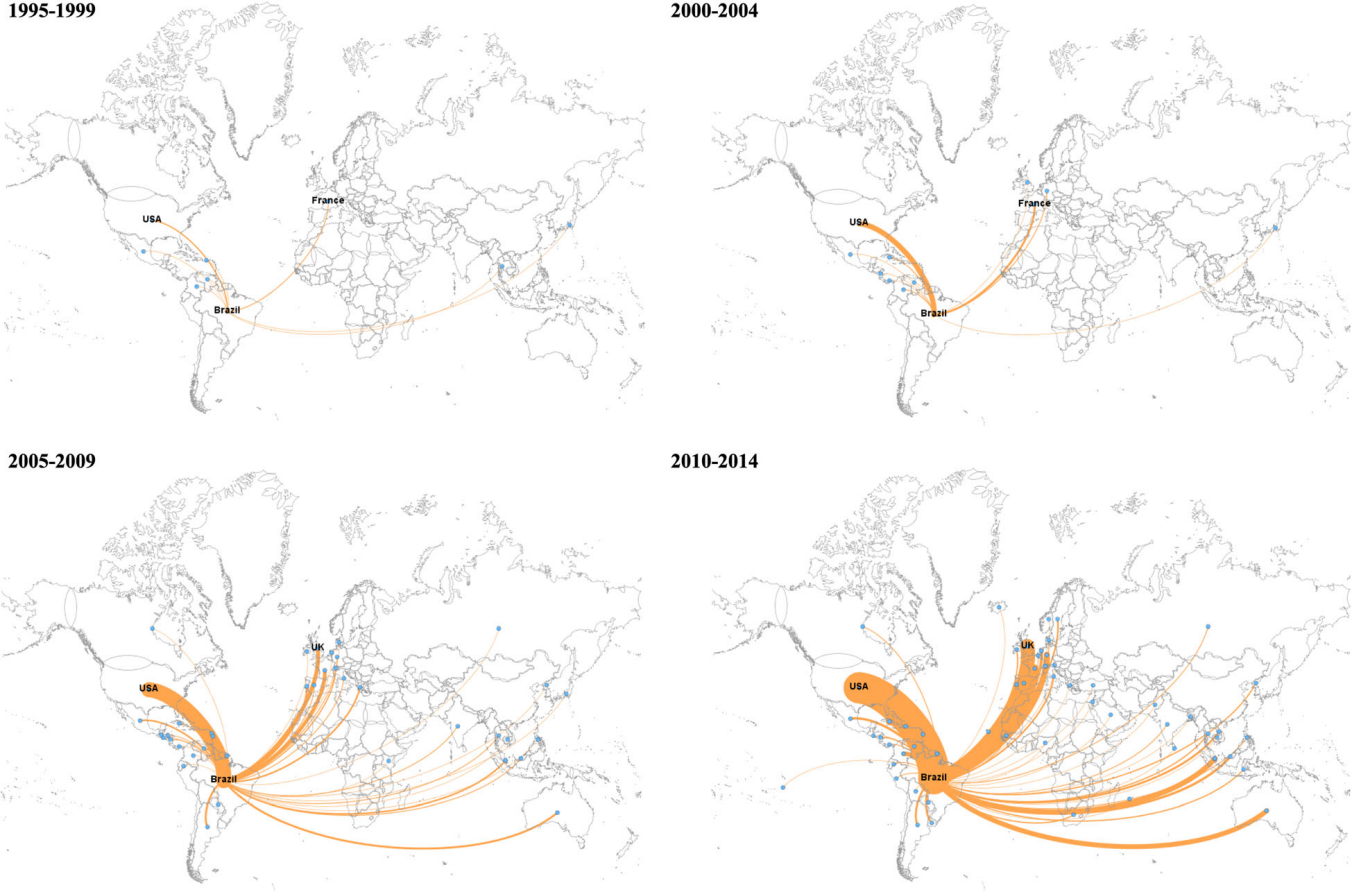
International collaboration in dengue research networks involving Brazilian organisations. Country links were mapped based on the affiliations of the authors of published papers. Each node represents one country and two countries were considered connected if their authors shared the authorship of a paper. The thickness of links indicates the frequency of collaboration between two nodes. Countries that have strongest collaborations with Brazil were named

Collaboration with North American and French organisations occurred more frequently in the first and second periods. From 2005 to 2014, organisations from the United Kingdom surpassed French institutions (Fig. 5).

Throughout the whole 20-year period, the most frequent Brazilian partners in international collaborations were the Johns Hopkins University (USA), the Pasteur Institute (France) and the University of Pittsburg (USA).

## Discussion

The application of SNA to generate information for policymaking processes in NTDs is an emerging field [28]. It has been used in policy planning for disease elimination/eradication [11], for mapping the interface between research and technological development [5], describing historical collaborations and their evolution [29, 30], and for prioritisation of research efforts [13, 14].

In this study, the longitudinal evaluation of dengue research networks identified structural and organisational patterns of Brazilian collaborative efforts. The integration of three databases offered a comprehensive representation of the Brazilian dengue research community and a good approximation of the research network structure and the key players involved.

Our findings suggest that Brazilian research organisations were embedded in highly connected networks strengthened through the years. The continuous increase in collaboration, more pronounced from 2004 onwards, can be related to a more active role of the Ministry of Health in defining and supporting research priorities since 2003. Previously, the limited engagement of the Ministry of Health in linking health research and the National Health Policy resulted in a gap between the production of scientific knowledge and the health needs of the population [31]. The funding availability after 2009 also matched the expansion of national and international networking presented in this article. A booster in dengue research funding occurred in 2009, when 15 collaborative projects involving 58 national and 19 international institutions were selected to study the dynamics of infection and disease control [17]. Dengue outbreaks from 2010–2013 in Brazil could also explain the increasing interest and trend in research collaboration, particularly on strategies for transmission control. The establishment of the Brazilian Program of National Institutes of Science and Technology in 2008 probably influenced the latest period of analysis. The program mobilised the top frontier research groups as nation-wide research networks, five of them on dengue-related topics.

Vector-related research, the most frequent theme of research in the past 15 years, addresses an important research need and a current challenge in dengue control. Implementation of routine vector control continues to be difficult and costly, and the development of resistance to insecticides is another emerging problem [32]. This has also been the most recent topic in international collaborations. This trend in scientific output reflects the fact that vector control is still the sole way of reducing transmission.

The CI consolidated information on four different centrality measures. Low CI values indicated organisations that had themselves a large number of connections (degree) and were related to the most connected institutions (eigenvector). These organisations were also expected to quickly obtain and disseminate information (closeness), most likely controlling knowledge flow in the network (betweeness). Being central in these networks means that they could have helped to both disseminate knowledge and facilitate access to resources and research opportunities, reducing the network vulnerability. As central organisations, they probably had a vital role in maintaining the connection between the overall research network and in ensuring that less well-connected or peripheral organisations gained access to new knowledge and information, as suggested by the reduced connectivity seen when they were excluded from the network.

The increase in dengue international research collaboration reflects to some extent the global increase in scientific collaboration [33]. The rapid geographical expansion of dengue could have fostered this cooperation [15]. International cooperation allows access to local knowledge and better understanding of disease transmission, diagnostics and morbidity dynamics in endemic settings. At the same time, developing countries’ scientists can benefit from the access to facilities, funding, equipment and networks that are often limited in their own countries. The Brazilian “Science Without Borders” program may have contributed to the increase in the internationalisation of research efforts. Launched in 2011 by the Council for Scientific and Technological Development, the program aimed at increasing STI Brazilian competitiveness through the exchange and international mobility of students.

Although Brazilian dengue research has remarkably evolved into a more collaborative, knowledge-intensive and international network, apparently this was not associated with a reduction in disease incidence. During the reviewed period, dengue incidence and outbreak intensity have steadily increased. This fact suggests a limited translation of research and development efforts into public health solutions.

Although there is still limited evidence regarding the effectiveness of SNA-based policies, the results presented herein have value in informing policymakers that (1) the Brazilian dengue research network is potentially very effective in knowledge generation, sharing and diffusion, suggesting a strong research capacity; (2) there is a small proportion of private companies involved in the research network, indicating the near-absence of collaboration with the academic sector in dengue research and development; (3) the central organisations identified herein are potential sources of information on technology trends and new partnerships, relevant for strategic decisions on investments; and (4) the central organisations involved in the latest network (2010–2014) were all located in the Southeast region of Brazil, which is the most academically developed and the most affected by dengue outbreaks. These organisations could facilitate the development and implementation of innovations at the regional level, reinforcing local health services.

The importance of networking to the quality and advancement of science has not always been recognised by national research programs, which tend to emphasise individual research investment in detriment of policies to support and foster networks. As research and product development for dengue and other NTDs are conducted in several centres around the world, research networks can be an efficient way of identifying gaps, synergies and using resources with benefits beyond national borders. STI policymakers should ensure that national scientists are part of networked systems to promote collaboration, optimise resources, potentiate results and avoid competition. Incentives to international collaboration should be included in national STI strategies and policies so that the local science base can benefit from the intellectual and financial leverage of international partnerships. Mobility grants from national and international funding agencies can be an effective instrument to encourage research collaboration.

Different perspectives regarding research collaboration could have been explored. We recognise the limitation of using co-authorship data as an indicator of scientific collaboration knowing that not all collaborative efforts result in publications, and not all co-authored papers necessarily imply collaboration in the form of knowledge sharing. Still, it is assumed that, in most cases, co-authorship indicates an active cooperation between partners beyond the simple exchange of material or information.

## Conclusions

During the period 1995–2014, Brazil has expanded considerably its national and international collaboration in dengue research, showing a shift from traditional public health towards basic biomedical and vector control research. The Brazilian research network in dengue has proven to be potentially very effective in knowledge generation, sharing and diffusion, maintained by key central institutions, but with limited engagement of the private sector.

The ultimate impact of networking on the capacity for product, process and services innovation and its translation into public health actions is yet to be evaluated. As for future direction for research it would be of interest to identify individual researchers who are most likely to sustain scientific productivity and networking and contribute to bridging the translational gap in dengue research. Such leading authors are expected to be important opinion makers and could assist in guiding STI policy and the promotion of research for public health and development.

SNA proved to be a valuable tool for mapping collaboration structures, research outputs, processes and network evolution. Along with other research and development indicators, this method can strengthen a knowledge platform to inform future policy, planning and funding decisions.

## Additional files

Additional file 1: Table S1. Dengue research networks excluding international organisations. (DOCX 12 kb)
Additional file 2: Matrix S1. Institutional network dataset (1995-1999). (CSV 3 kb)
Additional file 3: Matrix S2. Institutional network dataset (2000-2004). CSV 17 kb 
Additional file 4: Matrix S3. Institutional network dataset (2005-2009). (CSV 85 kb)
Additional file 5: Matrix S4. Institutional network dataset (2010-2014). (CSV 240 kb)
Additional file 6: Table S2. List of paper titles and corresponding macro research themes (1995-2014). (XLSX 88 kb)

## Abbreviations

NTD: Neglected Tropical Disease
S&T: Science and Technology
SciELO: Scientific Electronic Library Online
SNA: Social Network Analysis
STI: Science, Technology and Innovation
WoS: Web of Science
