# Supplementary material for: Dengue research networks: building evidence for policy and planning in Brazil
Source: Health Res Policy Syst. 2016 Nov 8;14:80. doi: 10.1186/s12961-016-0151-y (PMC5101674; doi:10.1186/s12961-016-0151-y)
Supplement: Additional file 1: Table S1. — Dengue research networks excluding international organisations. (DOCX 12 kb) [file 12961_2016_151_MOESM1_ESM.docx]

**Table S1: Dengue research networks excluding international organizations**

| **Indicator** | **1995-1999** | **2000-2004** | **2005-2009** | **2010-2014** |
| --- | --- | --- | --- | --- |
| **Number of nodes (organizations)** | 24 | 71 | 148 | 215 |
| **Number of links** | 36 | 94 | 305 | 616 |
| **Number of components** | 4 | 16 | 17 | 12 |
| **Giant component size** | 87.5% | 71.8% | 85.1% | 93.5% |
| **Average degree** | 3.42 | 3.49 | 4.69 | 6.10 |
| **Average clustering coefficient** | 0.688 | 0.687 | 0.709 | 0.690 |
| **Average path length** | 2.62 | 3.33 | 2.74 | 2.81 |
